# Immunomodulatory and Antitumor Effects of a Novel TLR7 Agonist Combined with Lapatinib

**DOI:** 10.1038/srep39598

**Published:** 2016-12-21

**Authors:** Ningning Gao, Jingjing Zhong, Xiaodong Wang, Zhenchao Jin, Wang Li, Yu Liu, Yuwen Diao, Zhulin Wang, Wenqi Jiang, Guangyi Jin

**Affiliations:** 1 National-Regional Key Technology Engineering Laboratory for Synthetic Biology of Medicine, Shenzhen University, Shenzhen 518060, People’s Republic of China; 2 Cancer Research Center, Shenzhen University, Shenzhen 518060, People’s Republic of China; 3 Department of Pharmacy, School of Medicine, Health Science Center, Shenzhen University, Shenzhen 518060, People’s Republic of China; 4 Sun Yat-sen University Cancer Center, Guangzhou 510060, People’s Republic of China; 5 Conjugenix company of Shenzhen, Shenzhen 518063, People’s Republic of China.

## Abstract

As new treatment approaches, both immunotherapy and targeted treatments have been used in the clinical treatment of cancers. These therapies are different from traditional surgery, chemotherapy and radiotherapy. Use of a combination of immunotherapy and targeted treatments may improve tumor clearance. We investigated the feasibility of combining tyrosine kinase inhibitors (TKIs, targeted drugs) and SZU-101 (a novel TLR7 agonist synthesized by our laboratory). Thirteen different TKIs were combined with or without SZU-101 and studied to determine their effects on immunocytes. On the basis of the distinctive results, lapatinib and sunitinib were selected for further tumor-inhibition investigation and determination of the underlying mechanism. Interestingly, we found lapatinib to work better with SZU-101, enhancing tumor clearance *in vivo*, without affecting the TLR7-NF-κB pathway activated by the TLR7 agonist in mouse spleen lymphocytes and bone marrow dendritic cells (BMDCs).

Owing to the limited efficacy and strong side effects of traditional cancer therapy, innovative therapies, such as targeted therapy and immunotherapy, are urgently needed^1^,^2^. Targeted therapies have shown high response rates and improved survival in tumor patients, but their application is impaired by the limited duration of their effects, owing to acquired resistance^3^. Immune responses have been studied at the molecular and cellular level, and promising treatments, such as checkpoint inhibitors, CAR-T, and cancer vaccines, have been developed^4^,^5^. These immunotherapy strategies have been associated with durable responses but may have limited efficacy in some patients. Thus, there is a growing interest in combination therapy to take advantage of both immunotherapy and targeted therapy^6^.

Our laboratory has focused on cancer immunotherapy, particularly, on the development of small Toll-like receptors (TLRs) agonists to activate the innate immune system against cancer. TLRs are fundamental elements of the immune system, which facilitate innate and adaptive immunity. The distinct molecular components of invading pathogens are recognized by these receptors and activate certain TLR pathways. TLR ligands control the activation of antigen-presenting cells by triggering their maturation program. Thus, agonists of TLRs have been considered as potential therapeutic agents for cancer^7^. Among them, TLR7/8 agonists are able to boost the immune system^8^, thus resulting in the induction of effective antitumor responses and long-lasting clearance of tumor cells^9^. Imiquimod is a ligand of TLR7 that has been used for the treatment of malignant tumors of the skin. Topical treatment with imiquimod (Aldara 5% cream) has been found to be clinically effective for both primary skin tumors and cutaneous metastases^10^. Recently, we have reported the synthesis of a novel TLR 7 agonist, SZU-101, and its use in combination with doxorubicin for cancer therapy. This combination induced durable tumoricidal effects in a murine model of T cell lymphoma^11^. We have also found that conjugating OCT4 protein to this agonist produces a vaccine that effectively and safely prevents tumor growth in mice^12^. Furthermore, after its conjugation to a monoclonal gastric cancer 7 antigen mono-epitope, the TLR7 agonist elicits nonspecific antitumor responses and strengthens specific humoral and cellular immune responses^13^. SZU-101 stimulates innate immune cells to release high levels of cytokines and affects the frequency of intratumoral immune cell infiltration^14^.

Inspired by these results, we would like to investigate the combinative effects of the TLR7 agonist with targeted therapy agents, particularly, with receptor tyrosine kinase (RTKs) inhibitors. RTKs are essential elements in signaling pathways that transduce extracellular signals into intracellular compartments and consequently regulate cellular growth, differentiation, metabolism and motility^15^,^16^. To date, more than 20 RTKs inhibitors have been approved by the FDA^17^,^18^. Although RTK inhibitors are designed to modulate the RTK signal transduction pathway, intriguingly, the therapeutic efficacy of several RTK inhibitors have been reported to be at least partially mediated by the immune system^19^. However, some RTK inhibitors have also been reported to have contradictory effects on the immune system^20^,^21^.

To investigate the possibility of combining SZU-101 and TKIs in breast cancer therapy and to determine the effects of these RTK inhibitors on the immune system and their potential for combination with immunotherapy, we screened 13 kinds of RTK inhibitors and monitored the cytokine release in immunocytes that resulted from combined use of SZU-101 and TKIs. The results indicated that lapatinib and sunitinib played a different role in combination with SZU-101, which were further confirmed by tumor growth inhibition assays in a tumor bearing mouse model. Finally, the antitumor mechanisms of SZU-101 and TKIs on TLR7/NF-κB pathways were investigated to evidence the efficiency of these combinations.

## Results

### *In vitro* cytokine release after combined administration of SZU-101 and TKIs

**Figure 1:**
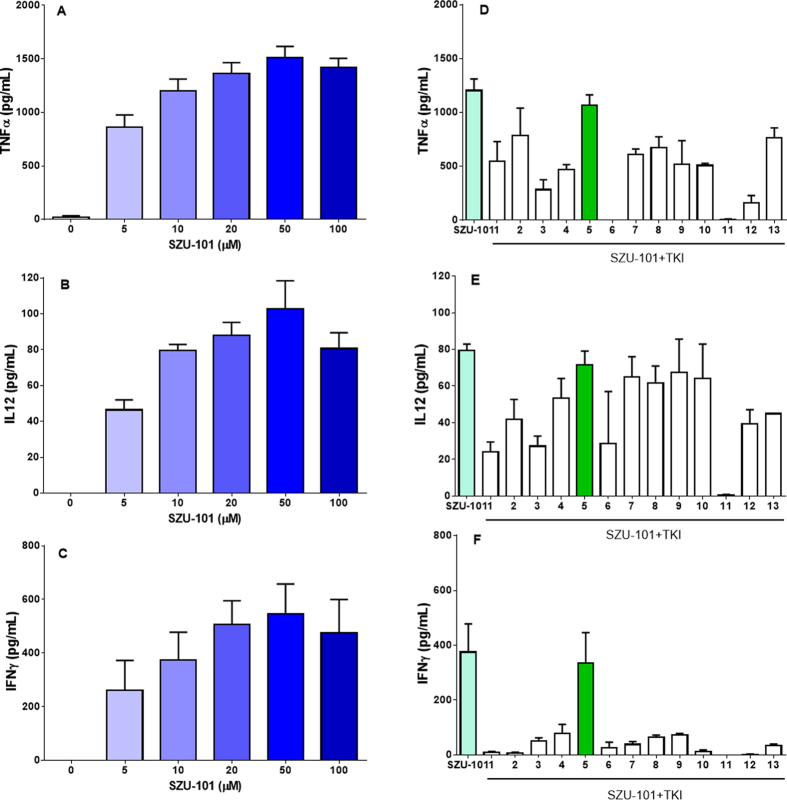
Cytokine induction in mouse spleen lymphocytes by the TLR7 agonist with/without TKIs. Mouse spleen lymphocytes were separated and incubated with SZU-10 at a concentration of 5, 10, 20, 50, or 100 μM for 24 hours. Cytokine (TNF-α, IL-12 and IFN-γ) induction was analyzed by ELISA (**A–C**). Mouse spleen lymphocytes were also treated with SZU-101 mixed with various TKIs (separately) at the indicated concentrations (10 μM SZU-101, 2 μM TKIs 3/5/6, 5 μM other TKIs). The induction of cytokines (TNF-α, IL-12 and IFN-γ) was analyzed by ELISA 24 hours later (**D–F**).

**Figure 2:**
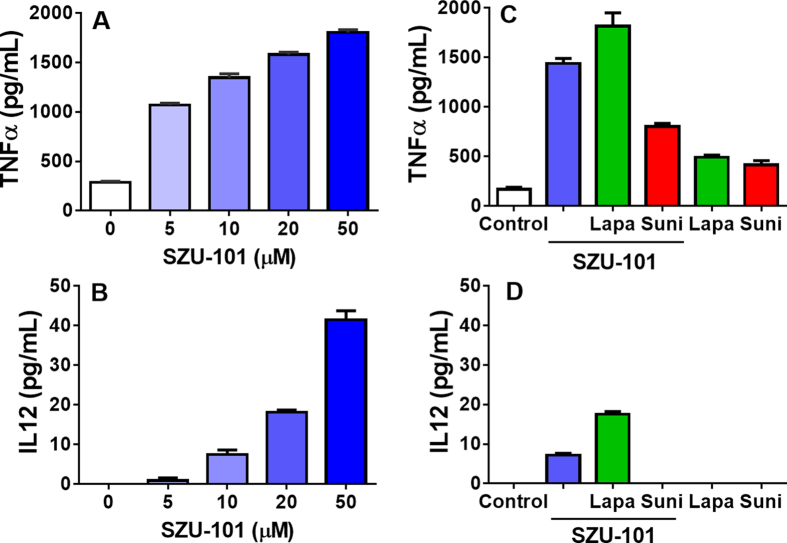
Cytokine induction in mouse BMDCs by the TLR7 agonist with/without TKIs. After separation and culture, BMDCs were incubated with SZU-10 at a concentration of 5, 10, 20, or 50 μM for 24 hours. Cytokine (TNF-α, IL-12) induction was analyzed by ELISA (**A,B**). Mouse BMDCs were also treated with SZU-101 mixed with lapatinib or sunitinib at the indicated concentrations (10 μM SZU-101, 2 μM lapatinib, 5 μM sunitinib). Induction of cytokines (TNF-α, IL-12) was analyzed by ELISA 24 hours later (**C,D**). (Suni, sunitinib; Lapa, lapatinib).

Thirteen different TKIs were used in this study: vandetanib (#1), nilotinib (#2), dasatinib (#3), imatinib (#4), lapatinib (#5), ceritinib (#6), gefitinib (#7), erlotinib (#8), ibrutinib (#9), pazopanib (#10), sunitinib (#11), crizotinib (#12) and sorafenib (#13). Most of the TKIs exerted inhibitory effects on 4T1 cells, as shown by a CCK-8 assay, after 24 h treatment. The cell growth inhibition (relative to the untreated control) showed a dose-dependent increase in the range of 5–20 μM (Figure S1), whereas, the cytotoxic effect on immunocytes was weak at the indicated concentrations (2 μM for the TKIs 3/5/6, 5 μM for the other TKIs) (Figure S2). To examine the effects on the production of certain critically necessary cytokines, mouse spleen lymphocytes were exposed to compounds at the above concentrations. Cytokine release was determined with ELISAs detecting IFN-γ/TNF-α/IL-12 in spleen lymphocytes and TNF-α/IL-12 in BMDCs after 24 h treatment with SZU-101. As shown in Fig. 1, SZU-101 treatment increased the release of cytokines IFN-γ, TNF-α and IL-12. When the various TKIs were used in combination with SZU-101, only lapatinib (#5) did not affect the stimulation of the TLR7 agonist in spleen lymphocytes, whereas the other TKIs inhibited the levels of cytokines induced by SZU-101. Cytokine assays were further performed in mouse BMDCs treated with 10 μM SZU-101, 2 μM lapatinib (#5) and 5 μM sunitinib (#11). Similarly to the results of spleen lymphocytes, there was no adverse effect of SZU-101 and lapatinib on the cytokine release levels of TNF-α and IL-12 in BMDCs (Fig. 2). However, sunitinib exerted an inhibitory effect on the SZU-101-induced activation of BMDCs.

### Tumor growth inhibition by SZU-101 and TKIs (lapatinib or sunitinib) *in vivo*

**Figure 3:**
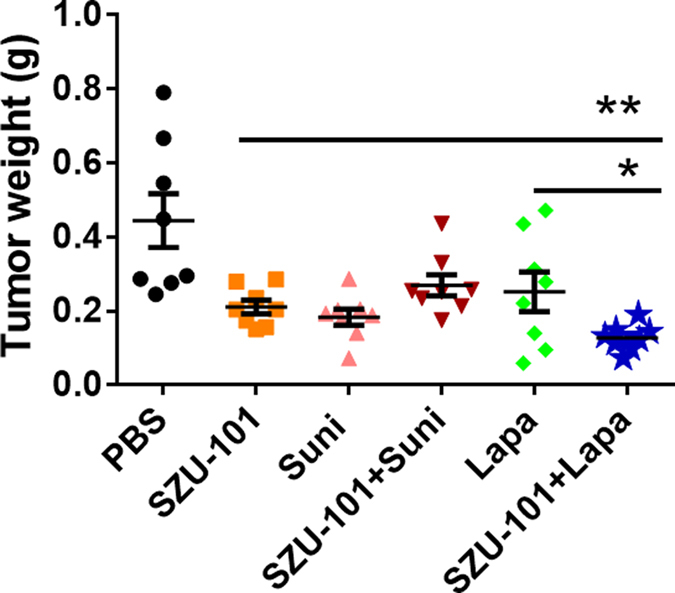
Therapeutic effectiveness of combined treatments on tumor-bearing mice. One week after inoculation with 2 × 10^5^ 4T1 cells, BALB/c mice were administered i.p. PBS only or PBS containing SZU-101 (5 mg/kg i.h. every 3 days), lapatinib (40 mg/kg i.p. every day), sunitinib (10 mg/kg i.p. every 2 days), SZU-101+lapatinib (two drugs used together) or SZU-101+sunitinib (two drugs used together). After 20 days of drug treatment, all mice were sacrificed, and the tumors were removed and weighed. (*p < 0.05, **p < 0.01, one-way ANOVA) (Suni, sunitinib; Lapa, lapatinib).

The effects of SZU-101 and TKIs on mouse breast cancer growth inhibition were further examined *in vivo* in a BALB/c mouse model inoculated with 4T1 cells. After 1 week of inoculation, all of the mice grew visible tumors of approximately 200 mm^3^, The mice were randomly distributed into six groups and then treated with SZU-101, TKIs or a combination of the agonist and a TKI. After 21 days of therapy, tumor weights had decreased in the mice exposed to SZU-101, lapatinib or sunitinib alone, compared with the PBS negative control (Fig. 3). Notably, the combination of SZU-101 and lapatinib resulted in a further shrinkage of tumor weight compared with that in the SZU-101 group (*p* < 0.01) or lapatinib group (*p* < 0.05), whereas the combination of SZU-101 and sunitinib, compared with either agent alone, had an inhibitory effect. Similarly, the indexes of tumor weight per mouse body weight of mice treated with a combination of SZU-101 and lapatinib were also minimal (Figure S3).

### Activation of TLR7/NF-κB pathways by combined treatment with SZU-101 and TKIs (lapatinib or sunitinib)

**Figure 4:**
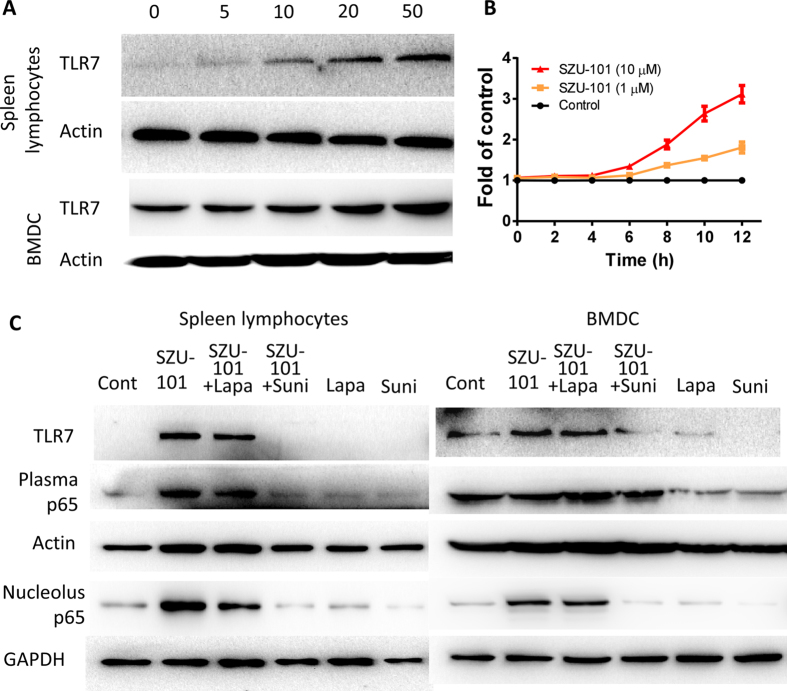
SZU-101 combined with/without TKIs mediated TLR7-NF-κB activity in mouse spleen lymphocytes and BMDCs. (**A**) Mouse spleen lymphocytes and BMDCs were treated with SZU-101 (5, 10, 20, or 50 μM) for 24 hours. After the treatment, cells were lysed, and cellular TLR7 levels were determined using immunoblotting. (**B**) Hek-Blue hTLR7 cells were treated with SZU-101 (1 or 10 μM) for 12 hours. The cells were tested with a Hek-Blue detection kit every 2 hours. The final optical density at 620 nm was normalized to the control group. (**C**) Mouse spleen lymphocytes and BMDCs were treated with SZU-101 (10 μM), lapatinib (2 μM), sunitinib (5 μM) and the combinations of SZU-101 and TKI. After the treatments, cells were lysed, and TLR7 and p65 levels were determined using immunoblotting.

It was important to confirm that SZU-101 activated immunocytes through the TLR7-associated pathway. Mouse spleen lymphocytes and BMDCs were exposed to SZU-101 for 24 hours, and the cell lysates were collected and subjected to western blotting. As expected, SZU-101 exhibited a dose-dependent activation of TLR7 expression in both spleen lymphocytes (5, 10, 20 and 50 μM) and BMDCs (5, 10, 20 and 50 μM) (Fig. 4A). Furthermore, we introduced a TLR7-NF-κB reporter system in HEK-293 cells and treated the cells with SZU-101. We found that the reporter was activated after a 4-hour incubation, and increased reporter activity of up to 3-fold was consistently induced by 10 μM SZU-101, which directly activated the NF-κB reporter system (Fig. 4B). This activation by SZU-101 was dose- and time-dependent. Similar trends were also evident in the cytokine assays, as shown in Figs 1 and 2. Higher concentrations of SZU-101 led to higher levels of these cytokines, thus suggesting that stimulation of the TLR7 pathway in immunocytes had indeed occurred. We also confirmed that the activation pathway was unrelated to TLR4 and high-mobility group protein B1 (HMGB1). SZU-101 did not significantly elicit HMGB1 secretion, and the TLR4 signaling inhibitor CLI-095 did not affect the activation induced by SZU-101 in mouse spleen lymphocytes and BMDCs (Figures S5 and S6).

Most importantly, the effects of SZU-101 and the TKIs on the TLR7 pathway were exhibited in spleen lymphocytes and BMDCs (Fig. 4C). SZU-101 induced a distinct increase of TLR7 expression in spleen lymphocytes, but the TKIs lapatinib and sunitinib had little effect. TLR7 remained unchanged when treatment with SZU-101 compared with SZU-101 and lapatinib combination, whereas sunitinib specifically inhibited the SZU-101-induced TLR7 expression. In addition, an increase in the nuclear p65 level after SZU-101 treatment suggested that SZU-101 stimulated the NF-κB pathway by increasing p65 translocation from the cytoplasm to the nucleus. Notably, the introduction of lapatinib or sunitinib exerted markedly different effects when it was combined with SZU-101. Similar results were obtained in BMDCs, in that SZU-101, rather than the TKIs, elevated the TLR7 expression, whereas sunitinib still degraded SZU-101-induced TLR7. In the NF-κB pathway, SZU-101 promoted translocation of the p65 protein to the nucleus, a result similar to our previously findings^22^. Moreover, sunitinib, instead of lapatinib, strongly depressed the SZU-101-mediated activation of the NF-κB pathway by interfering with p65 transcription in BMDCs. There was another evidence that the TLR7-NF-κB reporter was activated similarly by SZU-101 with/without lapatinib, whereas sunitinib suppressed the activation of SZU-101 at a certain degree (Figure S4).

## Discussion

The immune system, particularly in the tumor microenvironment, has recently been shown to play a crucial role in modulating tumor progression and the response to therapy. The majority of tumors have evolved mechanisms to evade immune responses. Recently, antibodies that target checkpoints have already demonstrated significant promise in clinical trials^23^, such as ipilimumab, which targets cytotoxic T-lymphocyte antigen 4 (CTLA-4) and the antibodies pembroluzimab^24^ and nivolumab^25^, which target programmed death receptor 1 (PD-1). Although these immune checkpoint therapies have shown durable responses^26^, they are effective in only a fraction of patients^27^. There is a growing trend to rationally combine immunotherapy with other therapies, such as radiation, cryotherapy, chemotherapy and targeted agents^27^,^28^.

Targeted drugs, such as tyrosine kinase inhibitors (TKIs), have been extensively investigated. For example, the epidermal growth factor receptor (EGFR) inhibitor erlotinib has shown efficacy in patients with acute myeloid leukemia, a type of cancer in which EGFR is not expressed^19^. The fusion oncogene EML4-ALK, which is found in 5% of NSCLC patients, is inhibited by crizotinib with a response rate of 65%^29^. However, patients treated with these targeting agents eventually develop resistance that leads to disease progression. In addition, these TKIs are not free of side effects. For example, the EGFR-specific small-molecule compounds erlotinib and gefitinib may cause a broad spectrum of adverse effects on skin and hair^30^.

Efforts to combine immunotherapy and TKIs have begun to take advantage of the duration of immunotherapy and the response rate of targeted therapy. Intriguingly, these TKIs may have off-target effects on the immune system and may thus affect their therapeutic efficacy positively or negatively. For example, sunitinib and sorafinib have been reported to decrease the levels of infiltrating regulatory T (Treg) cells and myeloid-derived suppressor cells (MDSCs) in patients with renal cell carcinoma (RCC)^31^,^32^ and to facilitate the development of antitumor Th1 responses^33^. In contrast, some TKIs may cause immunosuppression that is not favorable in cancer treatment. For example, dasatinib has been reported to inhibit lymphocyte-specific protein tyrosine kinase (LCK), overall favoring immunosuppression^34^. Nilotinib inhibits antigen-specific CD8^+^ T cell proliferation at pharmacological concentrations^35^.

It has been reported that some TKIs have more contradictory effects on the immune system. For example, sorafenib reportedly inhibits an array of DC functions, including cytokine secretion, the expression of co-stimulatory molecules and T cell activation^33^. However, it decreases the level of infiltrating Treg cells and MDSCs in patients with RCC^31^ and thus activates the immune system. In addition, erlotinib boosts the expression and exposure of various ligands for activating NK cell receptors, hence increasing the susceptibility of malignant cells to NK cell-mediated cytotoxicity in lung cancer cell lines^36^. However, erlotinib also inhibits monocyte differentiation^37^, perhaps favoring the increase in circulating MDSCs that generally accompanies tumor progression. The precise immunomodulatory effects of erlotinib and gefitinib *in vivo* remain to be characterized.

The effect of TKIs administered as part of combination therapies on the immune system have also been reported. It has been demonstrated in previous reports that TKIs exert synergistic effects with chemotherapeutic (e.g., capecitabine, vinorelbine) and immunotherapeutic agents (e.g., trastuzumab). The combined use of lapatinib and trastuzumab (a humanized monoclonal antibody) results in synergistic antiproliferative effects in HER2-overexpressing cell lines^38^, and lapatinib is also useful in the treatment of trastuzumab-resistant breast cancer cells^39^. Ibrutinib, an irreversible inhibitor of Bruton agammaglobulinemia tyrosine kinase (BTK), induces complete remission only when it is combined with an intratumoral injection of a TLR9 agonist, CpG^40^. Nevertheless, few investigations have been performed on the drug interactions between TKIs and TLR7 agonists administered simultaneously to treat breast tumor, which was our focus in this study.

In the current study, SZU-101 (a TLR7 agonist) was shown to stimulate the immune system, thus resulting in high levels of IFN-γ, TNF-α and IL-12. When SZU-101 was co-administered with 13 clinically relevant TKIs, except for lapatinib, all the TKIs showed a negative effect on SZU-101-induced cytokine release. Furthermore, in a tumor-bearing murine model, we observed therapeutic responses that coincide with the *in vitro* results. After 20 days of mono-therapy with SZU-101, lapatinib, or sunitinib, similar decreases in tumor weight were observed. However, lapatinib and sunitinib exerted markedly different effects on the tumoricidal activity of SZU-101.

It is very important to understand how different TKIs can affect the immune system and to use that knowledge to guide choices of drug combinations. First, the effect of the small molecule SZU-101 on the TLR7 pathway was investigated. Next, Hek-Blue hTLR7 cells were used to determine the effect of the bioactivity of SZU-101 on the activation of the TLR7 pathway. The cells were obtained by co-transfection of the TLR7 gene and an inducible SEAP (secreted embryonic alkaline phosphatase) reporter gene into HEK293 cells. Stimulation of the TLR7 pathway led to activation of NF-κB and AP-1, which in turn induced the production of SEAP and a visible blue color in the detection medium. As expected, SZU-101 demonstrated duration-dependent increases of TLR7 activation at concentrations of 1 μM and 10 μM. Enhanced TLR7 expression in both spleen lymphocytes and BMDCs provided further evidence of SZU-101 bioactivity.

TLR stimulation leads to the dimerization of TIR (Toll/interleukin-1 receptor) domains and the recruitment of adapter proteins, thus ultimately resulting in the activation of transcription factors, including members of the interferon (IFN)-regulatory factor (IRF) family and nuclear factor-κB (NF-κB)^41^,^42^. Therefore, NF-κB signaling is a downstream pathway of TLR7 activation that plays a crucial role in the immune response. The NF-κB dimer resides in the cytosol in the resting state and associates with the inhibitor of κB (IκB) protein, which prevents NF-κB from binding active sites. IκBα is a prototypical member of the IκB family whose primary target is the p65/p50 heterodimer complex. Degradation of IκBα and concomitant release of p65 is the classic mechanism of the canonical NF-κB pathway^43^. It has been shown that antitumor activity is partially associated with the activation of immunocytes via the NF-κB signaling pathway^44^. Our results showed that SZU-101 promoted the expression of TLR7 and p65 in spleen lymphocytes and BMDCs, whereas sunitinib clearly countered the effects of SZU-101.With regard to lapatinib, this promotion induced by SZU-101 were evidenced by the translocation of p65 protein to the nucleus, especially in BMDCs. The regulation of the TLR7/NF-κB pathway in immunocytes provides a mechanistic account of the antitumor effects of the combined use of SZU-101 and TKIs.

In conclusion, to our knowledge, this is the first report on the antitumor effects of combination therapy using TKIs and a TLR7 agonist in breast cancer treatment. The effects of SZU-101 on TLR7 signaling stimulation and various TKIs on cell growth inhibition were confirmed. Interestingly, lapatinib and sunitinib had exactly opposite effects on the immunological enhancement and tumor reduction elicited by SZU-101. Study of the underlying signal transduction pathways in spleen lymphocytes and BMDCs suggested that lapatinib or sunitinib may affect the immune system through a TLR7 agonist-activated NF-κB pathway. We cannot rule out the possibility that lapatinib and sunitinib have different tyrosine kinase targets. Our results suggest that the TKIs may have entirely different effects on the innate immune system and thus different effects on clinical outcomes. Lapatinib, an approved drug for breast cancer treatment, did not negatively affect the immune system, as other TKIs in our study did, and may be further evaluated for combination immunotherapy to treat breast cancer.

## Materials and Methods

### Ethics statement

All animal studies were approved by the Shenzhen University Administration on Laboratory Animal Care. Animals were treated in accordance with Shenzhen University Animal Ethical and Welfare Committee (AEWC) guidelines.

### TKIs

Nilotinib, dasatinib, imatinib, lapatinib, gefitinib, erlotinib, pazopanib and crizotinib were purchased from Sigma-Aldrich (MA, USA), Vandetanib, ceritinib, ibrutinib, sunitinib and sorafenib were purchased from Aladdin (Shanghai, China).

### Cytokine assays

Cytokines were measured in both mouse bone marrow dendritic cells (BMDCs) and mouse spleen lymphocytes. BMDCs were generated as described previously^45^. Briefly, bone marrow cells from the femur and tibia of a BALB/c mouse were cultured at 37 °C for 6 days in X-vivo 15 medium (Lonza Walkersville, MD, USA) containing 10 ng/ml GM-CSF and IL-4 (Peprotech, CA, USA). Spleen lymphocytes were isolated from BALB/c mice by using Mouse Lymphocyte Separation Medium (Dakewe, Beijing, China), according to the supplier’s manual. Briefly, total splenocytes were isolated from mice. Lymphocytes were harvested and subjected to density gradient centrifugation. BMDCs and lymphocytes were then seeded into 24-well plates at a density of 5×10^5^ cells per well. Compounds were added at the indicated concentrations, ranging from 2 to 100 μM, and incubated for 24 h. Then, the culture supernatants were collected, and cytokine quantification of TNF-α, IFN-γ and IL-12 was performed using mouse TNF alpha, IFN gamma and IL-12 p70 ELISA Ready-SET-Go reagent sets (eBioscience, San Diego, USA), according to the manufacturer’s instructions. Briefly, an ELISA plate was first coated with the capture antibody overnight at 4 °C and then filled successively with block solution for 1 h. Next, assay dilution (blank), standards, and samples (culture supernatant) were incubated for 2 h, and this was followed by incubation with antibody detection for 1 h at room temperature. Finally, substrate and stop solution were added to each well, and the optical density was measured at 450 nm with a spectrophotometer (BioTek, Winooski, USA).

### Tumor growth inhibition studies

This study was approved by the Laboratory Animal Ethics Committee of Shenzhen University. Four-week-old female BALB/c mice were purchased from the Medical Laboratory Animal Center (Guangzhou, Guangdong, China). All mice were housed under constant laboratory conditions, with a 12 h light/dark cycle and in specific-pathogen-free conditions and were fed with water and food *ad libitum.* After being acclimated for 1 week, each mouse was inoculated subcutaneously in the right side of the back with 2 × 10^5^ 4T1 cells. After 1 week, all mice grew visible tumors of approximately 200 mm^3^ and were randomly distributed into six groups of eight mice each: control (0.1 ml PBS i.p. every day), SZU-101 (5 mg/kg i.h. every 3 days), lapatinib (40 mg/kg i.p. every day), sunitinib (10 mg/kg i.p. every 2 days), SZU-101+lapatinib (two drugs used together) and SZU-101+Sunitinib (two drugs used together). After 20 days of treatment, all mice were sacrificed, and the tumors were removed and weighed.

### CCK-8 assay

Mouse 4T1 cells (ATCC, Manassas, USA), an animal model of stage IV human breast cancer cells, were cultured in DMEM medium supplemented with 10% fetal bovine serum, 100 U/ml penicillin and 100 μg/ml streptomycin in 25-cm^2^ culture flasks at 37 °C in a humidified atmosphere with 5% CO_2_. For the drug treatment experiments, cells were harvested from the culture during the exponential growth phase and then seeded into 96-well culture plates at 1 × 10^5 ^cells/ml in fresh medium. After overnight growth, the cells were treated with the compounds at the selected concentrations for 24 h. At the end of the drug treatment period, 10 μl of CCK-8 solution (Dojinodo, Shanghai, China) was added to each well of the culture plate. After a 90-min incubation, the optical density of living cells was obtained with a spectrophotometer at 450 nm (BioTek, Winooski, USA). The final optical density was normalized to the control group (medium).

### Hek-Blue assay

Hek-Blue hTLR7 cells (Invitrogen, Carlsbad, USA) were used for identification of the TLR7 agonist effect through the NF-κB signaling, owing to the stable expression of human TLR7 and the SEAP reporter. The cells were cultured on Hek-Blue^TM^ growth medium with 10 μg/ml Blasticidin, 100 μg/ml Zeocin and 100 mg/ml Normocin. After incubation with different doses of SZU-101, the cells were tested with a Hek-Blue detection kit every 2 h, according to the manufacturer’s instructions. The final optical density at 620 nm was obtained with a spectrophotometer (BioTek, Winooski, USA).

### Western blotting

Mouse bone marrow dendritic cells (BMDCs) and mouse spleen lymphocytes were isolated as described in the section on cytokine assays, and then, cells were treated with SZU-101, lapatinib or sunitinib. At the end of the drug treatment period, the cells were disrupted with Cell Lysis Buffer or a Nuclear and Cytoplasmic Protein Extraction Kit (Beyotime Institute of Biotechnology, Shanghai, China). The concentrations of the total protein, nuclear protein or cytoplasmic protein were determined with BCA assays. Equal amounts of protein samples were loaded onto a 10% SDS-polyacrylamide gel and then transferred to a microporous polyvinylidene difluoride (PVDF) membrane. Western blotting was performed using an anti-mouse TLR7 monoclonal antibody, anti-mouse p65 monoclonal antibody, anti-mouse GAPDH monoclonal antibody or anti-mouse β-actin monoclonal antibody (Cell Signaling Technology, Danvers, USA) and a horseradish peroxidase-conjugated secondary antibody (Cell Signaling Technology, Danvers, USA). Protein bands were visualized using Pierce ECL substrate (Thermo Scientific, Waltham, USA) and a FluorChem Q Western Blot Imaging System (ProteinSimple, Santa Clara, USA).

### Statistical analysis

Data were expressed as the means ± S.E.M for the indicated number of independently performed experiments. Dunnett’s t-test and one-way ANOVA were used for the determination of statistical significance, *p < 0.05, **p < 0.01, ***p < *0.001*.

## Additional Information

**How to cite this article**: Gao, N. *et al*. Immunomodulatory and Antitumor Effects of a Novel TLR7 Agonist Combined with Lapatinib. *Sci. Rep.* **6**, 39598; doi: 10.1038/srep39598 (2016).

**Publisher's note:** Springer Nature remains neutral with regard to jurisdictional claims in published maps and institutional affiliations.

## Supplementary Material

Supplementary Information
