# Supplementary material for: Immunomodulatory and Antitumor Effects of a Novel TLR7 Agonist Combined with Lapatinib
Source: Sci Rep. 2016 Dec 21;6:39598. doi: 10.1038/srep39598 (PMC5175151; doi:10.1038/srep39598)
Supplement: Supplementary Information [file srep39598-s1.doc]

**Supplementary Information**

**Immunomodulatory and Antitumor Effects of a Novel TLR7 Agonist Combined with Lapatinib**

Ningning Gao ^1,2,3,4 †^, Jingjing Zhong ^1,2,3†^, Xiaodong Wang ^1,2,3^, Zhenchao Jin ^1,2,3^, Wang Li ^1,2,3^, Yu Liu ^1,2,3^, Yuwen Diao^1,2,3^, Zhulin Wang ^5^, Wenqi Jiang ^2,4^*, Guangyi Jin ^1,2,3^ *

* Correspondence and requests for materials should be addressed to G.Y.J. (gyjin@szu.edu.cn) or W.Q.J. (wqjiang@szu.edu.cn)

^†^ Equal contributors

^1^ National-Regional Key Technology Engineering Laboratory for Synthetic Biology

of Medicine, Shenzhen University, Shenzhen 518060, People’s Republic of China.

^2^ Cancer Research Center, Shenzhen University, Shenzhen 518060, People’s Republic of China.

^3^ Department of Pharmacy, School of Medicine, Health Science Center, Shenzhen University, Shenzhen 518060, People’s Republic of China.

^4^ Sun Yat-sen University Cancer Center, Guangzhou 510060, People’s Republic of China.

^5^ Conjugenix company of Shenzhen, Shenzhen 518063, People’s Republic of China.

**Supplementary Information**

**Cytoxicity of TKIs on mouse breast tumor cell line 4T1.** 4T1 cells were seeded in 96-well plate at a density of 1×10^4^ cells per well. After 24 hours, various concentrations of TKIs were added for 24 hours and cell viabilities were assessed by CCK-8 assay (Figure S1). TKIs can inhibit the cell viability of mouse breast tumor cell line 4T1 [in various degree](http://dict.cn/in various degree).

Figure S1. Cell viabilities of 13 kinds of TKIs on 4T1 cell line. 4T1 cells were seeded in 96-well plate at a density of 1×10^4^ cells per well. After 24 h, various concentrations of TKIs were added for 24 hours and cell viabilities were assessed by CCK-8 assay. Thirteen different TKIs were vandetanib (#1), nilotinib (#2), dasatinib (#3), imatinib (#4), lapatinib (#5), ceritinib (#6), gefitinib (#7), erlotinib (#8), ibrutinib (#9), pazopanib (#10), sunitinib (#11), crizotinib (#12) and sorafenib (#13).

**Cytoxicity of TKIs on mouse spleen lymphocytes.** To examine the cytoxicity of TKIs, spleen lymphocytes were isolated from BALB/c mice using Mouse Lymphocyte Separation Medium, and seeded in 96-well plate at the density of 5×10^4^ cells per well. Then, lymphocytes were exposed to compounds at indicated concentrations (2 µM, 5 µM). After 24 h of incubation, cell viabilities were assessed by CCK-8 assay (Figure S2A). Similarly, BMDCs were generated as described previously, and seeded in 96-well plate at the density of 5×10^4^ cells per well. Compounds were added at the concentration of 2 µM and 5 µM. After 24 h of incubation, cell viabilities were assessed by CCK-8 assay (Figure S2B). Compounds hardly had any growth inhibitory effects on spleen lymphocytes and BMDC at 5 µM, except that three kinds of TKIs (#3, #5, #6) should be no more than 2µM.

Figure S2. Cytoxicity of TKIs on mouse spleen lymphocytes (A) and BMDC (B). spleen lymphocytes and BMDCs were seeded in 96-well plate at the density of 5×10^4^ cells per well. Compounds were added at the concentration of 2 µM and 5 µM. After 24 h of incubation, cell viabilities were assessed by CCK-8 assay

**Tumor weight per mouse body weight in Tumor growth inhibition studies.** After tumor-bearing and treatment with SZU-101, lapatinib and sunitinib as describe as “Materials and methods” section, mice were weight and then sacrificed. The tumors were separated followed by weighing also. The indexes of tumor weight per mouse body weight were calculated. Relative to their body weight, the tumor weight of combination treatment of SZU-101 and lapatinib were minium.

Figure S3. Tumor weight per mouse body weight in Tumor growth inhibition studies. After tumor-bearing and treatment with SZU-101, lapatinib and sunitinib as describe as “Materials and methods” section, mice were weight and then sacrificed. The tumors were separated followed by weighing also. The indexes of tumor weight per mouse body weight were calculated. (**p* < 0.05, ****p* < 0.001, one-way ANOVA) (Suni, sunitinib; Lapa, lapatinib)

**Activation of the combination of SZU-101 with lapatinib or sunitinib on TLR7-NF-κB reporter.** TLR7-NF-κB reporter (Hek-Blue hTLR7 cells) were cultured and detected as the manufacturer’s instructions. After treatment by SZU-101, lapatinib, sunitinib and the combination of agonist and TKI, the cells were tested with a Hek-Blue detection kit every 2 h from 0 to 12 hours, according to the manufacturer’s instructions. The final optical density of 620 nm was obtained with a spectrophotometer.

Figure S4. Activation of the combination of SZU-101 with lapatinib or sunitinib on TLR7-NF-κB reporter. After treatment by SZU-101 (10 µM), lapatinib (2 µM), sunitinib (5 µM) and the combination of agonist and TKI, the cells were tested with a Hek-Blue detection kit every 2 h from 0 to 12 hours. The final optical density at 620 nm was normalized to control group.

**SZU-101 did not elicit HMGB1 secretion significantly.** Mouse spleen lymphocytes and BMDCs were generated as described previously. After incubation with SZU-101, the culture supernatants were collected and HMGB1 quantification was performed using HMGB1 Detection Kit (Chondrex, Redmond, U.S.A.). SZU-101 could elicit slender production of HMGB1, which may be the paracrine results of other cytokines.

Figure S5. HMGB1 secretion of SZU-101 on murine spleen lymphocytes (A) and BMDCs (B). Lymphocytes and BMDCs were seeded in 24-well plates at a density of 5×10^5^ cells per well. SZU-101 were added at the indicated concentrations ranging from 5 to 50 µM and incubated for 24 h. Then the culture supernatants were collected and HMGB1 quantification was performed using HMGB1 Detection Kit.

**Disturbance of TLR4 signaling inhibitor CLI-095 on the activation of SZU-101.** Mouse spleen lymphocytes and BMDCs were generated as described previously. After incubation with SZU-101 with/without CLI-095 (Invitrogen, Carlsbad, U.S.A.), the culture supernatants were collected and cytokine quantification was performed by ELISA.

Figure S6. Disturbance of TLR4 signaling inhibitor CLI-095 on the activation of SZU-101. Murine spleen lymphocytes (A) and BMDCs (B) were seeded in 24-well plates at a density of 5×10^5^ cells per well. SZU-101 (from 5 to 50 µM) were added with/without CLI-095 (3 µM). The cytokine induction in culture supernatants was analyzed by ELISA 24 hours later.
